# Simultaneous structural and elemental nano-imaging of human brain tissue†

**DOI:** 10.1039/d0sc02844d

**Published:** 2020-08-10

**Authors:** Sian Genoud, Michael W. M. Jones, Benjamin Guy Trist, Junjing Deng, Si Chen, Dominic James Hare, Kay L. Double

**Affiliations:** Brain and Mind Centre and Discipline of Pharmacology, The University of Sydney Camperdown NSW 2050 Australia kay.double@sydney.edu.au; Central Analytical Research Facility, Institute for Future Environments, Queensland University of Technology Brisbane QLD 4000 Australia; Advanced Photon Source, Argonne National Laboratory Lemont IL 60439 USA; School of Biosciences, Department of Clinical Pathology, The University of Melbourne Parkville VIC 3010 Australia dominic.hare@unimelb.edu.au; Atomic Medicine Initiative, University of Technology Sydney NSW 2007 Australia

## Abstract

Examining chemical and structural characteristics of micro-features in complex tissue matrices is essential for understanding biological systems. Advances in multimodal chemical and structural imaging using synchrotron radiation have overcome many issues in correlative imaging, enabling the characterization of distinct microfeatures at nanoscale resolution in *ex vivo* tissues. We present a nanoscale imaging method that pairs X-ray ptychography and X-ray fluorescence microscopy (XFM) to simultaneously examine structural features and quantify elemental content of microfeatures in complex *ex vivo* tissues. We examined the neuropathological microfeatures Lewy bodies, aggregations of superoxide dismutase 1 (SOD1) and neuromelanin in human post-mortem Parkinson's disease tissue. Although biometals play essential roles in normal neuronal biochemistry, their dyshomeostasis is implicated in Parkinson's disease aetiology. Here we show that Lewy bodies and SOD1 aggregates have distinct elemental fingerprints yet are similar in structure, whilst neuromelanin exhibits different elemental composition and a distinct, disordered structure. The unique approach we describe is applicable to the structural and chemical characterization of a wide range of complex biological tissues at previously unprecedented levels of detail.

## Abstract 2

Structural and chemical characterisation of microfeatures in unadulterated Parkinson's disease brain tissue using synchrotron nanoscale XFM and ptychography.

## Graphical abstract

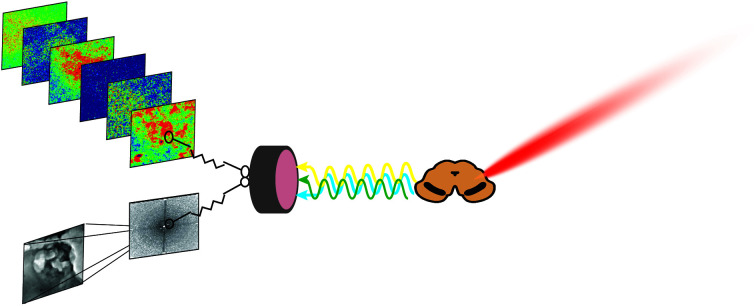

## Introduction

Multimodal imaging methods can provide information about the structural and chemical composition of biological specimens. Current methods require quantified regions of interest to be identified separately for each modality, and also necessitate varying sample preparation methods, both of which may compromise data quality. These limitations lower the specificity and sensitivity of these approaches, particularly for nanoscale imaging of complex *ex vivo* tissue samples. We present a method involving advances in synchrotron imaging technology and increasingly brilliant light sources, enabling multimodal nanoscale structural and chemical characterization of a wide range of complex biological tissues at previously unprecedented levels of detail in their native state. Here we apply this method to interrogate disease-associated microfeatures in fresh frozen human brain tissues.

Many neurodegenerative diseases are characterised by insoluble pathological structures within the central nervous system thought to represent products of molecular pathways involved in cell death.^1^ The formation of proteinaceous aggregates has been associated with biometal dyshomeostasis. Impaired or aberrant binding of the essential biometals Cu, Fe and Zn, in particular has been implicated in increasing the propensity of proteins to misfold and deposit within the central nervous system. Subcellular imaging of human post-mortem tissues provides insights into the biochemical mechanisms that underlie the formation of these pathological features in disease processes^2^ and is more widely applicable to other disease states and tissues.

We focused on three insoluble microfeatures involved in oxidative stress cascades of molecular neurodegeneration in Parkinson's disease (PD); Lewy bodies (LB), superoxide dismutase 1 (SOD1) protein deposits and neuromelanin (NM).^3^ LBs are amyloid-like deposits that are a defining pathological feature of PD. The presence of LBs in degenerating brain regions is essential for neuropathological diagnosis of PD,^4^ however LBs also form in non-degenerating PD brain regions.^5^ The 14 kDa synaptic peptide α-synuclein was identified as the primary constituent of LBs in 1997,^6^ and it is now widely believed that LBs represent the mature form of α-synuclein pathology, following oligomerization, aggregation, and deposition of this protein. α-Synuclein-associated toxicity in PD, and associated synucleinopathy disorders, are believed to primarily result from smaller oligomeric species and dysfunctional monomers of this protein.^5,7^ Nonetheless, as protein misfolding and deposition indicates impairment of normal protein structure and function, it is important to examine the structure and chemical composition of LBs to reveal the processes leading to their formation.

We also examined a separate population of proteinaceous aggregates we recently described in the degenerating substantia nigra (SN) of the PD brain which contain misfolded SOD1 protein.^8^ SOD1 is a homodimeric antioxidant protein that requires Cu and Zn binding in a 1 : 1 ratio for structural stability and normal enzymatic activity.^9^ Altered metalation of SOD1 is implicated not only in protein misfolding, but in a toxic gain-of-function of SOD1 protein that is associated with neuronal death.^3^ In the PD brain, SOD1 antioxidant activity is reduced specifically in the degenerating SN,^8^ a change thought to result from altered SOD1 protein metalation.^10^ This reduced activity likely increases SN neuronal susceptibly to oxidative damage, as this region already maintains a high level of basal oxidative activity resulting from dopamine metabolism and reactive by-products of mitochondrial activity.^11^ Examining the structure of SOD1 aggregates may also provide information regarding their formation in the PD brain.

Lastly, we analyzed deposits of NM, a biopolymer pigment comprised of dopamine oxidation products that gradually accumulate in neurons of the normal aging SN throughout the human lifespan.^12^ NM may play a role in biometal regulation in the human brain; NM purified from human brain exhibits a high affinity for metals,^13^ particularly pro-oxidant Fe at two binding sites (*K*_d_ (nM) = 7.18 ± 1.08; 94.31 ± 6.55).^14^ Like LBs, the role of NM in cell survival in PD has been debated.^15^ In the PD brain, the progressive loss of dopamine-producing neurons in the SN results in a depletion of NM levels in this region.^12^

Characterizing the elemental and structural composition of these microfeatures at nanoscale resolution can provide us with new insights into pathological pathways within the PD brain. Further, this technique can potentially uncover distinguishing features to aid in the label-free identification of LB and SOD1 aggregates, and NM, within the PD brain.

## Results and discussion

### Use of ptychography to identify microfeatures

Here we used the Bionanoprobe X-ray fluorescence microscopy (XFM) beamline at the Advanced Photon Source (see Materials and methods and ESI Fig. 2†) to examine distinguishing structural and elemental characteristics of insoluble deposits in chemically unadulterated brain tissue from five cases of sporadic PD (ESI Table 1†). The Bionanoprobe uses the same incident photon beam to simultaneously construct nanometer-resolution X-ray ptychography and XFM images without disturbing elemental distribution by repeated sample handling or potential observer effects.^16^

Ptychography uses scanning X-ray diffraction to reconstruct high sensitivity phase contrast images at a resolution limited by the measured diffraction signal, rather than the incident probe size or sampling interval.^17^ Compared to scanning transmission X-ray microscopy, ptychography provides higher resolution combined with high sensitivity phase contrast at hard X-ray energies, enabling the identification of structures that are otherwise unobservable through transmission, photon scatter or elemental XFM images. Furthermore, as high quality ptychography data can be collected at X-ray energies favorable to XFM, the two measurements can be combined into a single measurement rather than being collected sequentially.^18,19^ Prior examples of simultaneous XFM and ptychography used highly controlled systems such as cultured cells^20^ or multicellular model organisms.^21^ Here we report the first application of multimodal X-ray ptychography and XFM imaging to characterize distinct neuropathological features in complex, intact human brain tissue at nanoscale resolution.

### Native state chemical imaging of microfeatures

Native state chemical imaging aims to minimize *ex vivo* influences on the sample analyzed. Relatively weak electrostatic interactions between metal ions are particularly susceptible to chemical alterations, including tissue fixation, cell permeabilization, and changing pH.^22,23^ To prevent redistribution or loss of labile ions, fresh frozen tissue samples received from the tissue banks were subjected to minimal handling or processing. Pre-dissected tissues were provided on dry ice and were flash-frozen in liquid N_2_-cooled hexane to minimize freeze fracturing. Tissues were stored at −80 °C prior to cryosectioning at −12 °C (see Methods). Sections were mounted on Si_3_N_4_ windows, then air-dried in a dust-free environment and stored at room temperature until analysis. Measurements at the Bionanoprobe were taken at ambient temperature. Simultaneous collection of X-ray fluorescence spectra and phase contrast images allows measurement of unadulterated samples without risk of photoreduction and subsequent ‘observer effect’ from the ionizing incident hard X-ray beam.^24,25^

**Fig. 1:**
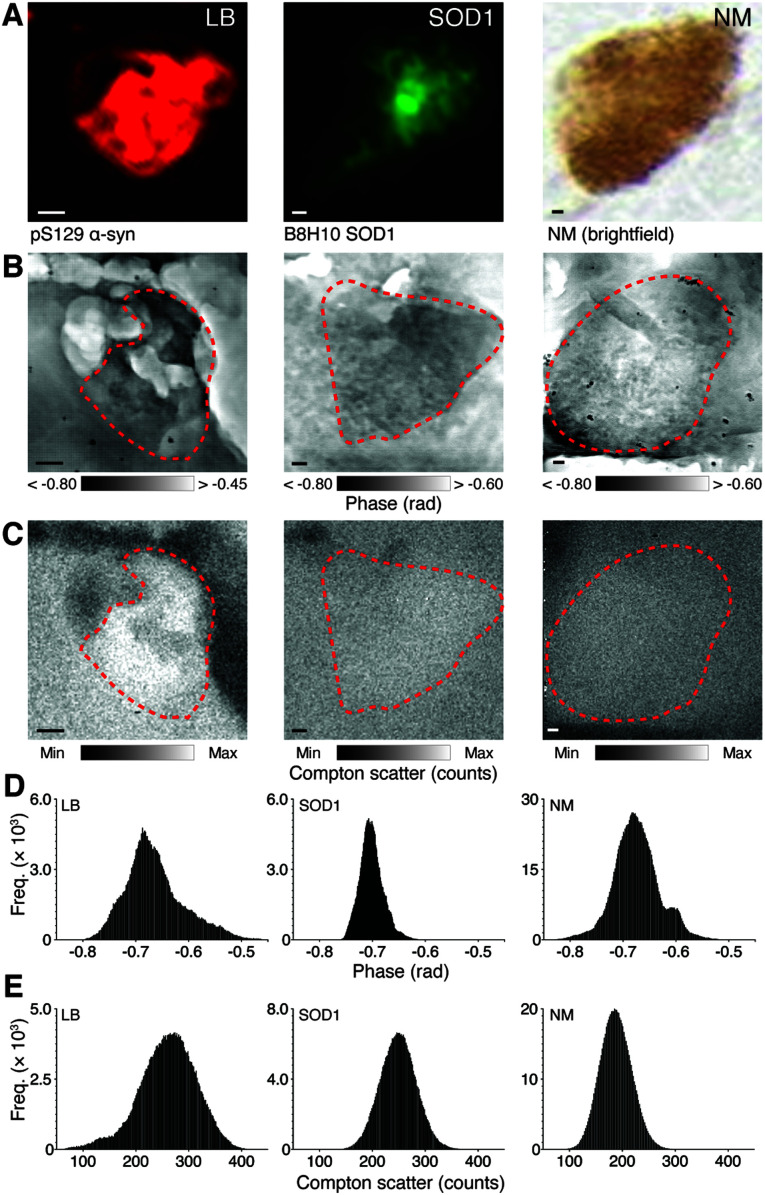
Identification of microfeatures using immunohistochemistry and corresponding nanostructure characteristics. (A) LBs and SOD1 aggregates in 20 μm fresh frozen SN sections are immunopositive for anti-human phosphorylated (pS129) α-synuclein, and anti-human misfolded B8H10 SOD1, respectively using immunofluorescence, while NM can be visualized in the native state using brightfield microscopy. Feature coordinates were used to identify corresponding regions of interest in facing sections mounted on Si_3_N_4_ windows. Simultaneous X-ray ptychography (B) and XFM (C) was used to construct spatial phase contrast (darker areas represent greater compositional diversity) and electron density (as Compton scatter, with lighter areas representing higher density) images of each identified feature. (D and E) Skewed histograms of phase contrast images reflect compositional diversity in the depth of structures, while normal frequency distributions from Compton scatter maps depict photon noise only. Scale bars = 1 μm. Representative images from scan IDs fly6 (LB), fly96 (SOD1), and fly67 (NM; see ESI Fig. 1† for corresponding elemental maps).

Far-field diffraction patterns collected by a PILATUS 300K pixel array detector placed 2.4 m downstream of the sample were used to reconstruct X-ray ptychography images with a pixel width of 13.5 nm and 182.25 nm^2^ area. X-ray fluorescence emission spectra were collected by an off-axis SII Vortex-ME4 and used to produce images of *K*_α_ and *K*_b_ emission peak energies with an 80 nm pixel width and 6.4 μm^2^ area (see ESI Data and Table 2†). Each neuropathological feature was identified using immunolabelling (LBs and SOD1 aggregates) or visualized using brightfield microscopy (NM; Fig. 1A). X-ray ptychography images of corresponding regions in adjacent facing sections (Fig. 1B) with spatial resolution approaching single-protein dimensions (ESI Fig. 3†) overcame the inability of inelastic X-ray fluorescence emission alone to define dense structures in brain tissue (Fig. 1C). High resolution imaging of phase change through the depth of 20 μm tissue sections using a 10 keV hard X-ray beam is necessary to define insoluble features without the likelihood of chemical alterations that arise when labelling with exogenous markers (Fig. 1D and E).

### LB and SOD1 demonstrate ordered structures

**Fig. 2:**
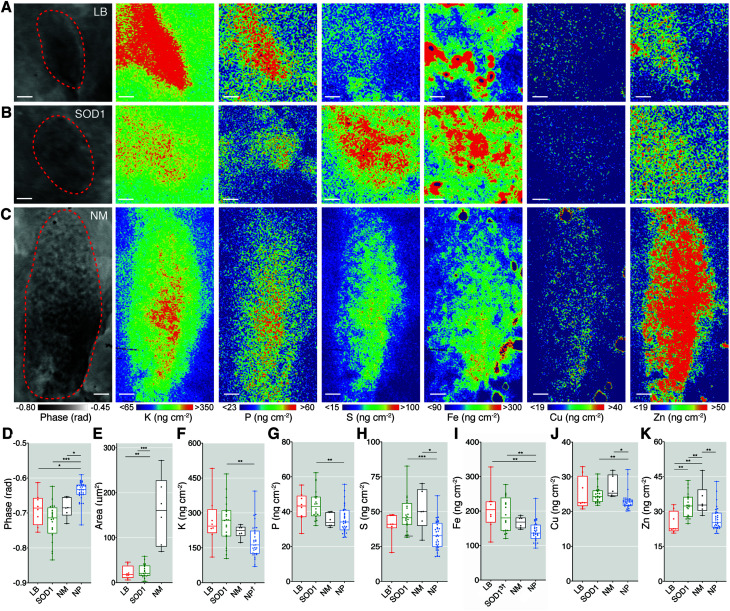
Quantitative X-ray fluorescence microscopy of LB, SOD1 aggregates and neuromelanin demonstrate distinct elemental fingerprints. (A–C) Phase contrast and XFM images of a representative LB, SOD1 aggregate, and NM deposit in the SN from the PD brain. (D) Greater average phase change per feature confirmed the distinctive compositional variation compared to surrounding neuropil (NP). (E) Proteinaceous LBs and SOD1 aggregates both had average areas of ∼24 ± 15 μm^2^, while NM granules occupied a larger and more irregular space (160 ± 77 μm^2^). (F–K) Spatial concentrations (*A*_mass_; as ng cm^−2^ from *K*_α_ and *K*_b_ emission spectra) of K, P, S, Fe, Cu, and Zn contrasted between features and the encircling neuropil matrix. Scale bars = 1 μm. Structures were sampled from 6–7 PD cases with multiple structures sampled from each case where time was allowed. Total structures analysed; *n*LB = 8, *n*SOD1 = 23, *n*NM = 6, *n*NP = 32, † = removed outlier. Box-plot graphs depict the median and quartile values as the central line and box boundaries. Whiskers represent min to max values. *p < 0.05, **p < 0.01, ***p < 0.001 based on Kruskal–Wallis (two-sided). Representative images from scan IDs fly25 (LB), fly98 (SOD1), and fly62 (NM).

XFM maps were upsampled using bicubic interpolation to 13.5 nm pixel widths and aligned to the ptychography images to extract elemental concentrations (*A*_mass_; areal mass as ng cm^−2^) within defined structural boundaries of individual features (Fig. 2A–C). Phase change within each insoluble deposit type was approximately 10% greater than the axons, dendrites and nerve fibers that make up the surrounding neuropil (Fig. 2D). The mean cross-sectional areas of LBs and SOD1 aggregates were both approximately 25 μm^2^ (Fig. 2E). Amorphous NM deposits were, on average, five times larger and more variable in size, as previously reported in large neuropathological studies of post-mortem PD brain (Fig. 2E).^26^ The width and normal distribution of phase change observed in LBs and SOD1 aggregates (Fig. 1D) suggest a similarly ordered aggregate structure in both pathologies. This is consistent with the amyloid-like properties reported in LBs; however interestingly SOD1 aggregates appear to exhibit non-amyloid properties.^8^ Nonetheless, SOD1 and LB aggregates both demonstrated inconsistent morphology, and both exhibited a wide range of sizes, (∼5–60 μm^2^), suggesting that they may be in varied maturation states of aggregate formation.

Disordered protein binding to the polymer may underlie the diverse and left skewed distribution observed in the phase change observed through granules of the NM pigment (Fig. 1D). This suggests a more disordered structure than that of LBs and SOD1 aggregates, and likely represents the gradual formation and maturation of NM in catecholamine-producing regions of the human brain from 3 years of age throughout the human lifespan.^26^

### Quantitative XFM demonstrates distinct elemental fingerprints

Six key elements (P, S, K, Fe, Cu and Zn) were simultaneously quantified in identified structures with high sensitivity and specificity using XFM (ESI Table 3†). The high spatial resolution enabled the detection of small perturbations in these elements unable to be identified using previous methods.

LBs in chemically unperturbed tissue contained ∼40% more Fe than both neuropil and SOD1 aggregates, and ∼30% less Zn than SOD1 and NM deposits, but levels of other elements were unchanged compared with other tissue compartments (Fig. 2F–K). SOD1 aggregates were richer in K, P, Fe, and Cu compared to the tissue matrix. Both SOD1 aggregates and NM were enriched in S, likely indicative of cysteine residues in SOD1 and sulfide moieties in the pheomelanin component of NM. High levels of K associated with insoluble deposits are likely indicative of electrostatic interactions that may seed initial aggregation of proteins,^27^ and diffusion of labile K^+^ from the cytosol and into NM deposits. Full descriptive statistics of each region of interest are presented in ESI Table 3.† After correction for possible self-absorption effects on elemental X-ray emission energies,^28^ most measured analytes exhibited enrichment within the denser protein aggregates and NM.

The binding of certain elements to α-synuclein including the biometals Cu, Fe and Zn, is suspected to potentiate oligomerization and neurotoxicity,^29^ although most studies have examined binding characteristics *in vitro*. Natively purified human α-synuclein from blood, brain, and peripheral tissue shows no evidence of significant interactions between the peptide and Cu, Fe, or Zn ions.^30^ Proteomic analyses of isolated LBs demonstrate these pathological inclusions consist of up to 300 proteins, including α-synuclein and small amounts of SOD1.^31,32^ The proteins contained in SOD1 aggregates in PD are yet to be fully described but are likely to include a range of biomolecules. NM is comprised of a pheomelanin core with a surface covering of eumelanin.^33^ In addition to the polymer backbone, the pigment appears to contain adducts of over 1000 identified proteins.^34^

As many of these proteins also bind various elements, elemental levels were normalized to aggregate density to indicate whether observed enrichment in these neuropathologies was merely a result of increased materials. Following density correction, elemental perturbations compared to surrounding neuropil can therefore be assumed to arise due to an altered binding propensity of molecules in aggregates.

### Impaired Cu-binding of SOD1 aggregates in the PD brain

The aggregates of wild-type SOD1 protein we recently identified in the PD brain^8^ are similar to inclusions that occur in degenerating motor neurons in approximately 20% of familial amyotrophic lateral sclerosis (ALS) cases linked to approximately 200 different mutations to the *SOD1* gene. Mature wild-type SOD1 normally binds one Cu and one Zn ion per monomer (1 : 1 ratio) to form a functional and stable homodimer.^9^ Reduced Zn^2+^ and Cu^+/2+^ binding to SOD1 plays a crucial role in the misfolding of this protein, and subsequent neurotoxicity in *SOD1*-associated fALS.^3^ Altered Zn^2+^ and Cu^+/2+^ binding to wild-type SOD1 is also suggested to be involved in similar pathogenic pathways in sporadic ALS,^35^ consistent with aggregation of wild-type SOD1 in PD.^36^

**Fig. 3:**
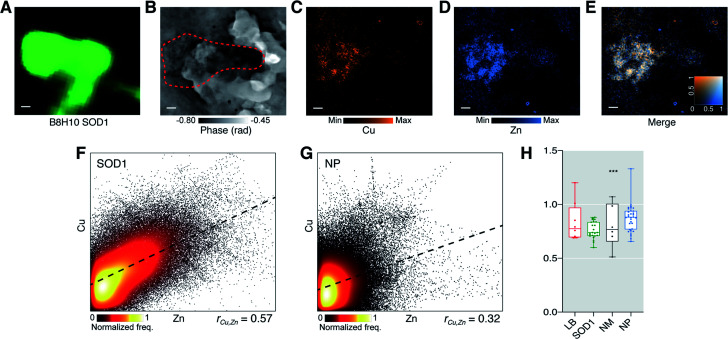
SOD1 aggregates in the PD brain have an altered Cu : Zn ratio suggestive of impaired Cu binding. SOD1 aggregate identified with (A) immunohistochemistry using anti-SOD1 (B8H10), (B) ptychography and (C–E) and XFM. SOD1 aggregates were analyzed for deviation in Cu : Zn ratio from a hypothetical mean 1 : 1 stoichiometry of holo-SOD1 dimers. (F and G) At true pixel size of 80 nm there was a strong positive spatial correlation between Cu and Zn within SOD1 aggregates but not in neuropil (NP). (H) A highly specific 26% decrease in Cu : Zn (*W* = −276; 95% confidence interval −0.288, −0.165; Wilcoxon signed rank test) was observed in the analyzed population of SOD1 aggregates. Representative images from scan IDs fly26 (SOD1). Scale bars = 1 μm. Structures were sampled from 6–7 PD cases with multiple structures sampled from each case where time was allowed. Total structures analysed; *n*LB = 8, *n*SOD1 = 23 *n*NM = 6, *n*NP = 32. Box-plot graph depicts the median and quartile values as the central line and box boundaries. Whiskers represent min to max values. *p < 0.05, **p < 0.01, ***p < 0.001 based on Kruskal–Wallis (two-sided).

We examined the ratio of Cu to Zn in SOD1 aggregates (Fig. 3) to determine if altered metal stoichiometry associated with SOD1 misfolding and aggregation *in vitro* (ref. 37) is present in chemically unadulterated SOD1 inclusions in human PD brain tissue. Eighty-three percent of Cu-containing pixels within SOD1 aggregates colocalized with Zn but only 69% of Zn-containing pixels colocalized with Cu, indicating the presence of other Zn-associated molecules (Mander's overlap coefficient). Spatial Cu and Zn location within identified aggregates were moderately correlated (*r*_Cu,Zn_ = 0.57; Fig. 3F), and no clear spatial correlation between Cu and Zn in surrounding neuropil was observed (*r*_Cu,Zn_ = 0.32; Fig. 3G). SOD1 aggregates likely contain other proteins that may bind these metals however, consistent with the hypothetical premise of SOD1 as the primary constituent protein, these data indicate most Cu and Zn are bound to the same structures within the 80 nm^2^ pixel dimensions, and that more Zn is present in the absence of Cu within the same area. The mean Cu : Zn ratio per SOD1 aggregate was consistently ∼25% lower than the 1 : 1 stoichiometry expected in fully metallated SOD1, whereas more variable and random average Cu : Zn ratios in LBs, NM, and neuropil were not (Fig. 3H). These data are consistent with *in silico* modelling showing Cu-deficient SOD1 has a greater propensity to aggregate^38^ and our previous findings of decreased activity of soluble wild-type SOD1 in the PD brain which is consistent with reduced Cu binding.^8^ Our results provide further evidence supporting altered post-translational modification of wild-type SOD1 in sporadic PD, leading to its aggregation.

## Conclusions

Simultaneous chemical and structural imaging at near diffraction limited resolution represents the next Frontier in nanoscale chemical characterization of complex biological systems. We demonstrate a method using simultaneous phase contrast X-ray ptychography and XFM to elucidate structural and chemical information in complex tissue samples not possible using either method in isolation. Applications of this method include interrogation of a diverse range of pathological features, and structurally diverse cellular organelles, in healthy and diseased tissues of any species. In the case presented here, we applied this multimodal imaging approach to examine the structural and chemical composition of distinct insoluble pathological features in human PD brain tissue.

## Materials and methods

### Human ethics approval and sample details

This study was approved by the University of Sydney Human Research Committee (approval number 2015/202). Human post-mortem brain tissue was obtained from the London Neurodegenerative Diseases Brain Bank at King's College London, and the Multiple Sclerosis and Parkinson's Tissue Bank at Imperial College London (United Kingdom). Fresh-frozen substantia nigra (SN) tissue samples were obtained from patients with idiopathic PD (*n* = 7 cases) confirmed by post-mortem examination according to standard diagnostic criteria.^39^ Subject demographics are listed in ESI Table 1.†

### Sample embedding and storage conditions

Fresh-frozen tissue blocks were shipped from the UK to Australia on dry ice and were immediately embedded in cryomolds using optimal cutting temperature compound (ProSciTech, QLD, Australia). Tissue was kept at a maximum temperature of −20 °C during embedding and were then flash-frozen by immersion in liquid N_2_-cooled hexane. Tissue was stored at −80 °C until required.

### Sectioning

Each tissue block was pierced 3 times with an acupuncture needle (38-gauge, 0.20 mm thickness) for orientation to match consecutive sections. All samples were sectioned at 20 μm using a CryoStar NX50 cryostat (ThermoFisher Scientific, Waltham, MA, USA) using C.L. Sturkey ‘Diamond’ PTFE-coated stainless-steel disposable microtome blades (ProSciTech, Australia). The cryostat chamber was set at −20 °C and the sample holder at −12 °C. The first section was used for immunohistochemistry (IHC) and was mounted on a Superfrost™ microscope slide (ThermoFisher Scientific, USA). The adjacent section was reversed and mounted on a Si_3_N_4_ window (5 × 5 mm, 200 nm film thickness, 200 μm frame thickness; Australian National Fabrication Facility, QLD, Australia) with the matching tissue face exposed for analysis.

### Immunostaining

Immunostaining procedures for SOD1 aggregates and LBs were adapted from (ref. 40) and recommended antibody supplier protocols. The first collected sections were post-fixed by 100% acetone at −30 °C for 10 min before drying for 20 min at room temperature. Sections were washed in 0.1 M PBS (pH 7.4) and peroxidases were quenched with 0.3% H_2_O_2_ in PBS for 20 min at room temperature. Sections were then washed again in 0.1 M PBS and blocked in 0.5% casein in PBS for 1.5 h at room temperature.

Sections were incubated overnight at 4 °C in conformation specific SOD1 primary antibody (1 : 80 SOD1 B8H10; Cat # MM-0070-P, Medimabs Inc, Montreal, Canada) in 0.5% casein and 0.1 M PBS. Sections were then washed in 0.1 M PBS and incubated in appropriate HRP-conjugated secondary antibody (1 : 1000 in 0.5% casein, Merck, Kenilworth, NJ, USA) for 2 h at room temperature. After another wash in 0.1 M PBS, sections were then incubated in Cy3 tyramide (*λ*_ex_ = 554 nm, *λ*_em_ = 568 nm; PerkinElmer, Waltham, MA, USA) for 10 min at room temperature and then washed in 0.1 M PBS. Sections were then probed phosphorylated α-synuclein primary antibody (1 : 150 α-synuclein phosphoserine 129 in 0.5% casein; Cat # ab51253 Abcam, Cambridge, UK), and appropriate HRP-conjugated secondary antibody (1 : 1000 in 0.5% casein, Merck, USA) and Cy5 tyramide (*λ*_ex_ = 650 nm, *λ*_em_ = 669 nm; PerkinElmer, USA). Slides were then cover-slipped in 80% glycerol.

### Optical and fluorescence microscopy

Immunolabelled tissue sections were imaged using an Olympus VS120 slide scanner (Olympus, Shinjuku City, Tokyo, Japan), using the red and Texas red channels for immunolabelled slides and using brightfield for fresh sections on Si_3_N_4_ windows. Corresponding images from facing sections were overlaid using OlyVIA 2.9 software (Olympus, Japan) and regions of interest were identified from fluorescent emission. Acupuncture needle marks were used to triangulate regions of interest from immunolabelled slides to identify the same regions on the Si_3_N_4_ windows. Pixel coordinates of aggregates and NM were recorded and later transferred to motor stage positions at the Advanced Photon Source.

### Bionanoprobe operating parameters

The Bionanoprobe is a hard X-ray nanoprobe located at 9-ID-B of the Advanced Photon Source, Argonne National Laboratory in Illinois, USA (see ESI Fig. 2† for end-station configuration).^16^ A 10 keV incident beam was focused by stacked Fresnel zone plate (70 nm outermost zone width) to a ∼100 nm spot on the sample. The scans were performed in fly-scan mode with an 80 nm pixel size and 50 ms dwell time per pixel. Total X-ray fluorescence emission spectra and far-field diffraction patters were collected simultaneously using a SII Vortex-ME4 and PILATUS 300 K pixel array detector, respectively. The Vortex detector was mounted at 90° to the incidence beam and the PILATUS detector was placed 2.4 m behind the sample with its detection surface perpendicular to the beam path. Pixel coordinates from OlyVIA 2.9 were directly imported into the Bionanoprobe sample stage using the integrated motor control system described in detail by Chen *et al.*, 2014.^16^

### X-ray fluorescence microscopy

Elemental maps were extracted at *K*_α_ and *K*_b_ emission energies. Spectrum from each pixel was analysed and quantified using a thin-film standard sample (RF8-200-S2453, AXO DRESDEN GmbH, Germany) using MAPS software^41^ where the areal mass (*A*_mass_: μg cm^−2^) was fitted for each measured element. Images were exported as 32-bit tiffs with units of *A*_mass_ and a pixel size of 80 nm for analysis using Fiji software (National Institute of Health, USA).^42^

### X-ray ptychography

Ptychograms were reconstructed with an iterative ePIE algorithm^43^ for 100 iterations using five orthogonal probe modes in a parallel GPU enabled environment.^44^ Expected phase value was calculated by modelling the tissue section as model protein (as C_30_H_50_N_9_O_10_S) with the assumption of a uniform density of 1.35 g cm^−3^.^45^ Phase change expected through the background tissue (neuropil) was calculated by obtaining the index of refraction (*δ* = 1.20 × 10^−6^)^46^ and using the formula:Phase = −*k* × *δ* × *z*where *z* = tissue thickness, *k* = 2π/*λ*, and *λ* = 9.6118 × 10^−11^.

The expected phase change was calculated as −1.57 radians and observed phase change in the supporting neuropil was −0.67. Following the correction of the arbitrary offset (0.90), area and density of reconstructed images were exported as phase (in radians) images with a pixel size of 13.5 nm.

### X-ray fluorescence microscopy and ptychography image registration

Ptychography and fluorescence data was aligned by initially scaling XFM images to match the resolution of the phase contrast images using bilinear interpolation before alignment using control point image registration in MATLAB v2019b (MathWorks, Natick, MA, USA). Registered data were exported as text images for analysis in Fiji.

### X-ray fluorescence microscopy image quantification

Images of *A*_mass_ (as ng cm^−2^) for data extraction were prepared in Fiji. Limit of detection (LOD) for each element was determined by locating holes in the tissue section correcting each pixel for the mean background *A*_mass_ + 3*σ*. High intensity spikes in *A*_mass_ attributed to nanoscopic contamination found mainly in transition metals were confirmed by inspection of image histograms. Using the ‘threshold’ tool, upper 1% were masked, designated as an undefined ‘not a number’ (NaN) unit, and subsequently excluded from analysis.

### Data extraction

Facing immunofluorescence (for pS129 α-synuclein and SOD1 B8H10) or brightfield microscopy photomicrographs were coarsely orientated with ptychographic images as a guide for freehand drawing of feature outlines as regions of interest (ROIs) in Fiji. Images were normalised by pixel-wise division of Compton scatter emission to correct for variation in surface density and self-absorption events.^47,48^ The ROI defined by the aligned ptychography image was then used to extract area and *A*_mass_ data from stacked XFM elemental maps.

### Colocalization analysis

Correlation analysis of Cu and Zn spatial maps was measured as Pearson's *r*, Costes' regression threshold, and Mander's overlap coefficients; performed in Fiji using the ‘Coloc 2’ plugin.

### Statistical analysis

Extracted LOD corrected XFM data were imported into SPSS Statistics v26 (International Business Machines, Illinois, USA) with matching subject demographic data. Outliers were defined by SPSS as ‘extreme values’ ≥3 × the interquartile range (or 2 standard deviations) and identified measurements were excluded. Normality was assessed using the D'agostino–Pearson omnibus test for Gaussian distribution. The non-parametric data were compared using the Kruskal–Wallis multivariate test for independent samples (two-tailed) with Tukey's *post hoc* correction for multiple pairwise comparisons. Effects of age, post-mortem interval, and sex were tested using bivariate correlation of variables. Statistical significance was reported according to the *α* = 0.05 value. A significantly different Cu : Zn ratio from 1 : 1 was inferred from a 95% confidence interval that did not span 1 (Wilcoxon signed rank test). Box and whisker plots were prepared in Prism v8.4.2 (GraphPad, San Diego, USA).

### Protein dimensions

Dimensions were calculated using the Inertia Axis Aligned Bounding Box method by the ‘Draw_Protein_Dimensions.py script in PyMOL. (Version 2.3.4; Schrödinger, USA) written by Pablo Guardado Calvo.^49^

## Conflicts of interest

Authors have no competing interests to declare.

## Supplementary Material

SC-011-D0SC02844D-s001
