# Supplementary material for: Simultaneous structural and elemental nano-imaging of human brain tissue
Source: Chem Sci. 2020 Aug 10;11(33):8919–27. doi: 10.1039/d0sc02844d (PMC8163372; doi:10.1039/d0sc02844d)
Supplement: SC-011-D0SC02844D-s001 [file SC-011-D0SC02844D-s001.pdf]

Electronic Supplementary Material (ESI) for Chemical Science.
This journal is © The Royal Society of Chemistry 2020

Electronic Supplementary Information

Simultaneous Structural and Elemental Nano-Imaging of Human Brain Tissue

*Sian Genoud, Michael W.M. Jones, Benjamin G. Trist, Junjing Deng, Si Chen, Dominic J. Hare, and Kay L. Double**

* kay.double@sydney.edu.au and dominic.hare@unimelb.edu.au

Table of Contents

List of Abbreviations 2

Supplementary Data 3

Supplementary Figures 4

Supplementary Fig. 1. Elemental maps of LB, SOD1 aggregate and neuromelanin in the PD brain 4

Supplementary Fig. 2. Bionanoprobe schematic 4

Supplementary Fig. 3. Structure and dimensions of protein aggregates and neuromelanin 5

Supplementary Tables 6

Supplementary Table 1. Subject demographics 6

Supplementary Table 2. APS Bionanoprobe scan identifiers 6

Supplementary Table 3. X-ray ptychography and fluorescent emission characteristics of human Parkinson's disease brain tissue 7

Supplementary References 8

List of Abbreviations

α-syn	α-synuclein
A_{mass}	areal mass
APS	Advanced Photon Source
ALS	amyotrophic lateral sclerosis
B8H10	misfolded human SOD1, clone B8H10
CCS	copper chaperone for SOD1
LB	Lewy body
LOD	limit of detection
NM	neuromelanin
NP	neuropil
PD	Parkinson’s disease
PDBe	Protein Data Bank in Europe
pS129	phospho serine129
rad	radians
SD	standard deviation
SEM	standard error of the mean
Si_3N_4	silicon nitride
SOD1	superoxide dismutase 1
SN	substantia nigra
XFM	X-ray fluorescence microscopy

Supplementary Data

Complete raw image data files can be accessed via PDImagingDataset.zip (private link). See Supplementary Table 2 for sample key.

Supplementary Figures

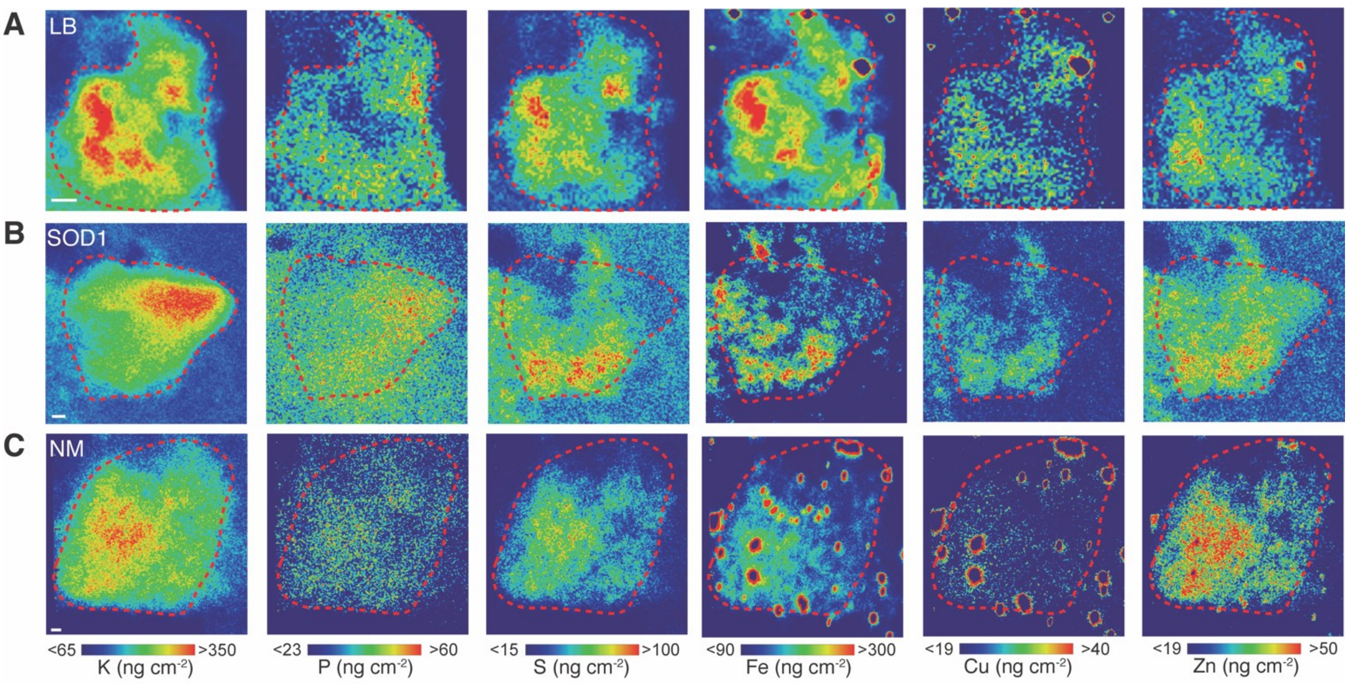

Supplementary Fig. 1. Elemental maps of LB, SOD1 aggregate and neuromelanin in the PD brain
XFM corresponding to immunohistochemistry and nanostructure images in Figure 1 of the main text. Representative elemental maps for A) LB B) SOD1 aggregate and C) neuromelanin of the six key elements K, P, S, Fe, Cu and Zn. Scale bars = 1µm. Representative images from scan IDs fly6 (LB), fly96 (SOD1) and fly67 (NM).

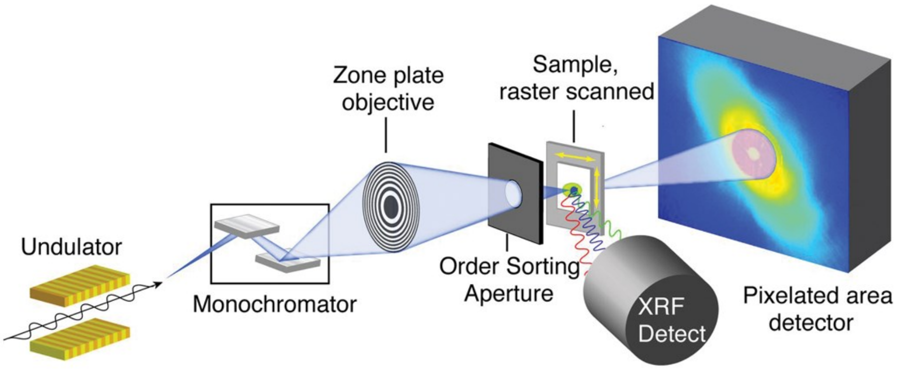

Supplementary Fig. 2. Bionanoprobe schematic
Schematic of the X-ray fluorescence and ptychographic imagine at the Bionanoprobe of the Advanced Photon Source. Reproduced from Deng et al (reproduced per Creative Commons Attribution 4.0 International License, 2017)[1].

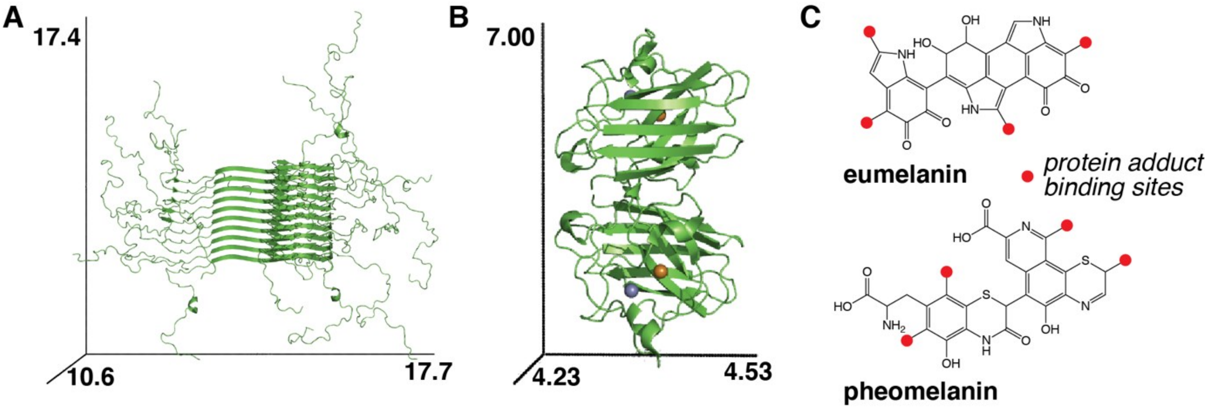

Supplementary Fig. 3. Structure and dimensions of protein aggregates and neuromelanin
a) Atomic-resolution structure of pathogenic α-syn fibrils (PDBe ID 2n0a[2]) b) Wild type holoSOD1 homodimer (PDBe ID 2v0a[3]). Protein structure and dimensions (nm) determined using PyMOL 2.3.3 (see Methods). c) Chemical structure of eumelanin and pheomelanin subunits of neuromelanin.

Supplementary Tables

Supplementary Table 1. Subject demographics

Reported PD cohort characteristics.

Case ID	Disease duration (y)	Age; onset (y)	Age; death (y)	PMI (h)[a]	Sex	Reported cause of death	Anti-parkinson medications
PD1	13	71	82	28	M	Bronchopneumonia, old age; PD	Levodopa
PD2	15	61	80	29	M	Urosepsis; PD	NR[b]
PD3	NR	NR	81	28	F	End-stage PD	NR
PD4	5	76	84	25	F	NR	Levodopa
PD5	NR	NR	89	54	F	NR	NR

[a] Post-mortem interval. [b] Not reported.

Supplementary Table 2. APS Bionanoprobe scan identifiers

Sample reference key for Bionanoprobe fly scan data and image identifiers[a].

Case ID	LB scan ID	SOD1 scan ID	NM scan ID
PD1	6, 22, 25	18[b], 19, 20, 23, 24, 26	-
PD2	-	45, 46, 78, 80	-
PD3	66, 68, 99	63[b], 64, 96, 98	62, 65, 67, 69, 70
PD4	106, 109	107, 108, 110	-
PD5	-	121	120
**Features imaged (*n*)**	8	23	6

[a] In archived (.zip) Supplementary Data as /fly<scan ID>/. [b] Multiple features in single fly scan area.

Supplementary Table 3. X-ray ptychography and fluorescent emission characteristics of human Parkinson's disease brain tissue
Limit of detection, sample size, mean, standard error of the mean (SEM), standard deviation (SD) and inter-sample min-max values for Lewy bodies (LB), SOD1 aggregates, neuromelanin (NM) and surrounding neuropil (NP).

Analyte	Limit of detection ($ng\ cm^{-2}$)[a]	Feature	*n*	Mean	SEM	SD	Min	Max
Area (μm^2)	-	LB	8	18.82	4.55	12.04	5.97	43.05
		SOD1	20	35.62	4.1	16.42	2.77	58.63
		NM	6	159.9	31.48	77.1	69.4	271.7
Phase (rad)	-	LB	8	-0.69	0.02	0.05	0.75	0.61
		SOD1	20	-0.74	0.13	0.05	-0.83	-0.66
		NM	6	-0.68	0.01	0.03	-0.73	-0.65
		NP	32	-0.64	0.01	0.03	-0.73	-0.59
Compton (counts)	-	LB	8	217.51	14.48	40.95	148.94	268.27
		SOD1	20	237.54	13.83	60.27	183.88	408.8
		NM	6	190.6	6.79	16.64	168.64	216.04
		NP	32	203.65	11.15	63.09	132.13	400.29
K ($ng\ cm^{-2}$)	66.9	LB	8	266.17	38.99	110.29	110.47	491.76
		SOD1	20	260.21	21.05	94.16	104.01	467.93
		NM	6	220.95	11.3	27.68	172.84	250.26
		NP	32	179.52	12.72	70.83	69.71	394.21
P ($ng\ cm^{-2}$)	23.4	LB	8	40.81	2.85	7.54	27.5	49.65
		SOD1	20	43.98	2.05	8.19	34.51	62.35
		NM	6	35.07	1.63	3.98	30.97	40.05
		NP	32	36.1	1.47	7.91	25.54	55.65
S ($ng\ cm^{-2}$)	15.6	LB	8	39.39	3.42	9.06	20.67	47.87
		SOD1	20	49.45	3.09	12.37	33.14	82.67
		NM	6	51.32	5.97	14.62	29.66	70.22
		NP	32	34.45	1.83	9.88	17.78	61.39
Fe ($ng\ cm^{-2}$)	93.3	LB	8	188.56	16.05	42.48	110.38	227.56
		SOD1	16	188.98	12.67	50.66	120.27	276.8
		NM	6	166.99	6.88	16.86	147.3	187.6
		NP	30	141.68	5.51	29.67	92.8	236.95
Cu ($ng\ cm^{-2}$)	19.2	LB	8	24.2	1.63	4.32	20.76	32.92
		SOD1	20	25.22	0.57	2.29	22.58	30.82
		NM	6	26.94	1.34	3.28	24.4	31.91
		NP	32	23.21	0.48	2.59	20.26	32.08
Zn ($ng\ cm^{-2}$)	19.6	LB	8	30.49	1.69	4.47	22.49	35.43
		SOD1	20	33.1	1.3	5.21	26.81	43.27
		NM	6	34.89	2.79	6.82	28.37	47.63
		NP	32	27.34	0.95	5.1	20.64	42.94

[a] Limit of detection (3σ + Si_3N_4 blank) as A_{mass} (in $ng\ cm^{-2}$) for measured elements.

Supplementary References

1. J. Deng, D. J. Vine, S. Chen, Q. Jin, Y. S. G. Nashed, T. Peterka, S. Vogt and C. Jacobsen, *Sci. Rep.*, 2017, **7**, 445.
2. M. D. Tuttle, G. Comellas, A. J. Nieuwkoop, D. J. Covell, D. A. Berthold, K. D. Kloepper, J. M. Courtney, J. K. Kim, A. M. Barclay, A. Kendall, W. Wan, G. Stubbs, C. D. Schwieters, V. M. Y. Lee, J. M. George and C. M. Rienstra, *Nat. Struct. Mol. Biol.*, 2016, **23**, 409-415.
3. R. W. Strange, S. Antonyuk, M. A. Hough, P. A. Doucette, J. A. Rodriguez, P. J. Hart, L. J. Hayward, J. S. Valentine and S. S. Hasnain, *J. Mol. Biol.*, 2003, **328**, 877-891.
